# Supplementary material for: Behavior classification: Introducing machine learning approaches for classification of sign-tracking, goal-tracking and beyond
Source: PLoS One. 2025 May 29;20(5):e0323893. doi: 10.1371/journal.pone.0323893 (PMC12121781; doi:10.1371/journal.pone.0323893)
Supplement: S1 Code — (DOCX) [file pone.0323893.s001.docx]

%import data in numerical values format (double) and make sure you

%import only one column/day of data at a time (so N x 1 instead of N x 6)

%you can either use individual days or mean-scores of the i.e. two last days

%this piece labels the PCA scores into three categories

%border values: -0.5 =>1 and 0.5 => 3

A = PCAdata;

str={'GT';'IN';'ST'};

B=interp1([-1,-0.5,0.5,1],[1,2,3,3],A,'previous');

%this creates a density function estimate based on the distribution of the data set

[f,xi] = ksdensity(PCAdata);

[f, xi, bw] = ksdensity(PCAdata);

disp(['Default bandwidth: ', num2str(bw)]);

%to visualize the density function

figure;

subplot(1,2,1);

plot(xi,f);

%deriving the f(x) curve

dfdx = diff(f)./diff(xi);

%this bit finds the maximums and minimums. Theoretically, we find the

%minimums by adding the abs function, which uses the absolute values to find

%the minimums. The peakLoc output gives the indices associated with appropriate

%value in xi. The cutoff values indices we are looking for are the second and

%third values in peakLoc (if the curve is binomial) We then go in xi to find

%the PCA score associated with indices of interest. ex; peakLoc xi = 22 and

%xi(22) = -0.45, which would be GT cutoff value.

[peakVal, peakLoc, peakWidth] = findpeaks(abs(dfdx));

[n_of_peakloc] = size(peakLoc);

if n_of_peakloc(2) == 4

%good distribution with 2 peaks

dfdx_cutoff(1) = xi(peakLoc(2)); %GT/IN

dfdx_cutoff(2) = xi(peakLoc(3)); %IN/ST

elseif n_of_peakloc(2) == 5

dfdx_cutoff(1) = xi(peakLoc(2));

dfdx_cutoff(2) = xi(peakLoc(4))

elseif n_of_peakloc(2) == 6

%good distribution with 3 peaks

dfdx_cutoff(1) = xi(peakLoc(2)); %GT/IN

dfdx_cutoff(2) = xi(peakLoc(5)); %IN/ST

else

%Careful the distribution may contain too many peaks

dfdx_cutoff = 0;

end

%applying the findpeaks function on the ksdensity output, and not the

%derivative, to find the peaks locations (stored in peakLocs_ks).

[peakVal_ks, peakLoc_ks, peakWidth_ks] = findpeaks(f,xi);

%This will give an array with the dfdx of all cases in the PCAdata

%-1 = GT, 0 = IN, 1= ST

[Y] = length(PCAdata);

if (n_of_peakloc(2) == 4 || n_of_peakloc(2) == 5 || n_of_peakloc(2) == 6)

for k = 1:Y(end)

if PCAdata(k,1) < dfdx_cutoff(1)

dfdx_out_array(k) = -1;

elseif PCAdata(k,1) > dfdx_cutoff(2)

dfdx_out_array(k) = 1;

else

dfdx_out_array(k) = 0;

end

end

else

dfdx_out_array = 0;

end

%the following part uses the k-Means cluster algorithm to group data into 3

%categories.Defining k allows to pick the number of clusters (we want3)

%The idx output is the groups that the algorithm associates observations with.

%We can use these labels (1,2,3) to visualize the cutoff values with the

%fitctree function.

%WARNING. The 1,2,3 labels are not necessarily equivalent to GT, IN and ST

%in that order. You need to check which label was provided to each group

%with the C output. In C, you will find 3 values [3,1] and each one of them

%is located on the associated label. If the value located at C(2,1)is equal

%to -0.40, it means that the associated label to the GT group is 2 in idx.

%Note that by providing Nx5 matrix for PCA data using the parameters of PCA

%score instead of the score itself, the kmeans runs 3 clusters on

%5-D data, data has to be numeric

[idx,C,sumd,D] = kmeans(PCAdata,3);

%Visualize the groups with the k-means cutoff.

% Y can be [1:1:N] where N = the number of observations in PCAdata

Y = [1:1:length(A)];

subplot(1,2,2);

gscatter(PCAdata,Y,idx,'rgb','osd')

%this bit is creating/using a decision tree to visualize class edges

%make sure that idx has the same number of observations than X

%otherwise it won't run, possible that NaNs were removed in idx but

%not in X

t = fitctree(PCAdata,idx,'PredictorNames',{'Score'});

view(t,'Mode','graph');
