# Supplementary material for: Behavior classification: Introducing machine learning approaches for classification of sign-tracking, goal-tracking and beyond
Source: PLoS One. 2025 May 29;20(5):e0323893. doi: 10.1371/journal.pone.0323893 (PMC12121781; doi:10.1371/journal.pone.0323893)
Supplement: S2 Fig — (DOCX) [file pone.0323893.s002.docx]

**Supplementary material**

# **Effect of Food Availability and the Validation Sample Characteristics**

# Two nonparametric one-way ANOVA tests (Kruskal-Wallis) were also carried out to assess the effects of food availability and sexes on PCA Index scores. The Kruskal-Wallis results from food restriction suggested a significant effect of food availability on PCA scores, with a small-medium effect size (*H* = 9.80, *p* = 0.002, ε² = 0.052). Furthermore, results from the Kruskal-Wallis test revealed that there were no significant differences between PCA Index scores of males and females (χ² = 0.00863, *p* = 0.926). The contradictory nature of the nonparametric and parametric results about the impact of food availability makes it challenging to draw clear conclusions from these analyses. However, it is worth noting that the line graph shown in (**Sup. Fig. 1)** contains appropriately constructed CI (difference- and correlation-adjusted 95% CI), therefore indicating a difference between scores of food-restricted and non-restricted male subjects, whereas this contrast is not evident in females.

# We conducted a mixed-design analysis of variance (ANOVA 2x2x6) on the PCA scores to evaluate if there were PCA Index scores differences based on the subjects’ sex, the food availability, and training day (**Sup. Fig. 2)**. Levene’s Test for homogeneity of variances showed uneven variances on Days 2 and 3 of training. Furthermore, sphericity assumptions were not met (*W* = 0.118, *p* < 0.01). The results revealed that only one factor significantly influenced the scores (*p* = 0.007), which was the training day.

# **
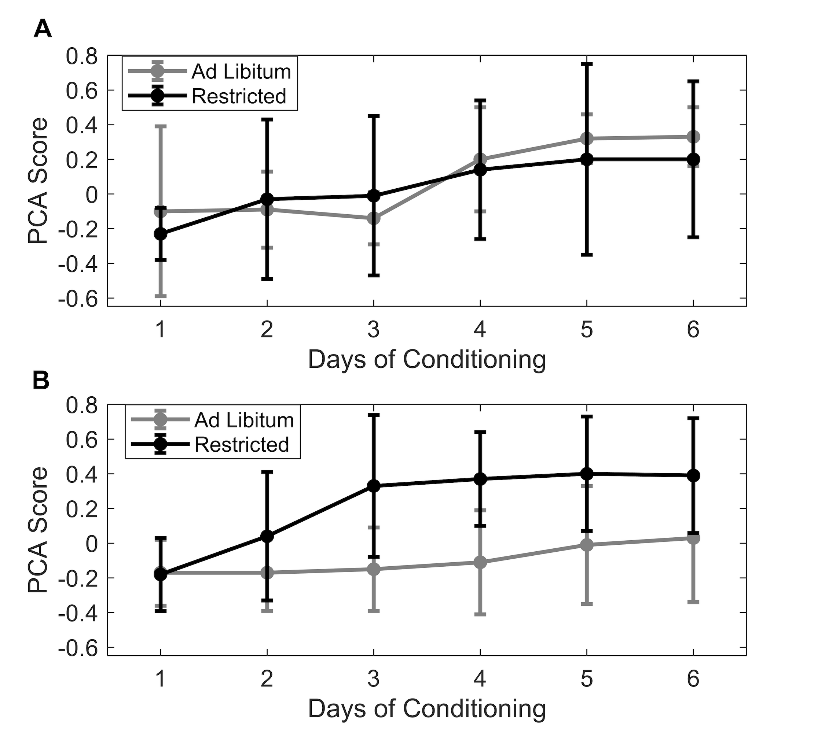
**

# **Sup. Fig. 1. Effect of food availability on male and female PCA Index scores per days of conditioning.**

# (A) Females. (B) Males. The circles illustrate the mean Pavlovian Conditioning Approach (PCA) Index scores obtained on each day of PCA conditioning, according to food availability. The black line illustrates subjects under a food restriction program, the gray line illustrates subjects given food ad libitum. The error bars depict difference- and correlation-adjusted 95% Confidence Intervals (CI), computed according to the method presented by Cousineau et al. (26). Food group differences appear in males, but not in females.


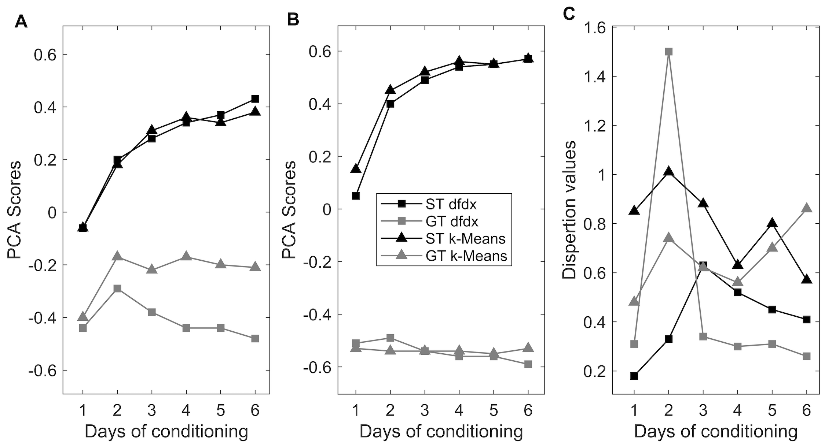


**Sup. Fig. 2*.* Cutoff values and dispersion of the *k*-Means and derivative methods in**

**the modeling sample.**

(A) Cutoff values for sign-trackers and goal-trackers extracted from the *k*-Means and the derivative (*dfdx*) methods. (B) Center points extracted by each method (centroids for *k*-Means and peak location for the derivative). (C) Dispersion around the group center (ST and GT groups). Both methods seem relatively stable after Day 4 of conditioning, indicating their validity for classifying scores after the 4^th^ measure.
